# Cisplatin-Induced Nephrotoxicity and HIV Associated Nephropathy: Mimickers of Myeloma-Like Cast Nephropathy

**DOI:** 10.1155/2017/6258430

**Published:** 2017-07-10

**Authors:** Muhammad Siddique Khurram, Ahmed Alrajjal, Warda Ibrar, Jacob Edens, Umer Sheikh, Ameer Hamza, Hong Qu

**Affiliations:** St. John Hospital and Medical Center, Detroit, MI, USA

## Abstract

Myeloma cast nephropathy is an obstructing disorder of renal tubules, caused by precipitation of Bence Jones proteins. Myeloma-like cast nephropathy (MLCN) has been reported in the literature to occur in various primary renal and nonrenal diseases. We present a series of three rare cases of cast nephropathy, two of which are HIV patients, and the third patient is receiving cisplatin-based chemotherapy. However, in all three patients plasma cell dyscrasia has been ruled out. A 30-year-old male was admitted to the hospital with facial cellulitis. The second patient is a 31-year-old male who presented with* Pneumocystis jiroveci* pneumonia. The third patient was treated with cisplatin-based chemotherapy for carcinoma. First two cases revealed foci of diffuse tubular dilatation containing hyaline casts and interstitial inflammatory infiltrate, in addition to globally sclerotic glomeruli with ultrastructural foot process fusion and mesangium expansion. The third case showed acute tubular injury and cast formation of irregular casts composed of amorphous or granular material of low density admixed with scattered high electron-dense globules. Myeloma-like cast nephropathy and true myeloma cast nephropathy pose similar destructive effects on renal parenchyma. This new pattern of HIV-related nephropathy should be considered in HIV patients with MLCN, once monoclonal gammopathy is ruled out.

## 1. Introduction

Myeloma cast nephropathy (MCN) is the most common pattern of renal injury found in plasma cell dyscrasia and more specifically multiple myeloma (MM). It commonly presents as acute renal injury, which prompts the physician to perform renal biopsy. The pathophysiology of MCN is precipitation of monoclonal immunoglobulins and Tamm-Horsfall glycoproteins in distal tubules and collecting ducts. The histologic hallmark of MCN is the presence of tubular casts, which appear eosinophilic and brittle and usually instigate an intense inflammatory response (cellular reaction) in kidney leading to acute renal failure (ARF); if untreated it can lead to irreversible damage [1, 2].

Myeloma-like cast nephropathy (MLCN) has been reported in the literature to occur in various primary renal and nonrenal diseases including some neoplastic processes, such as pancreatic carcinoma [3]. Some reports also suggested an association of MLCN with antimicrobial therapy [4].

Human immunodeficiency virus associated nephropathy (HIVAN) is the most common disease delineated in biopsy series of patients with HIV infection and renal disease [5, 6]. Two patterns of HIV kidney disease have emerged: HIV associated nephropathy (HIVAN) and HIV immune complex kidney disease (HIVCKD). HIVAN is suggested to be caused directly by HIV-1 infection, possibly via disruption of the normal homeostatic function of mature podocytes caused by HIV proteins Nef, Vpr, and Tat [7]. HIVAN itself is a collapsing glomerulopathy and the most common renal complication amongst patients with HIV-1 infection. HIVCKD is an immunoglobulin related glomerulonephritis. HIVCKD is characterized by immune complex deposition that includes complement, HIV-1 antigens, and reactive antibodies. Typically it is also associated with concurrent infections such as hepatitis C [8]. Immune responses are central to the development of HIVCKD, being associated with the concurrent infections and the deposition of HIV antigen antibody complexes within the glomerulus. For both HIVAN and HIVCKD, hyperplasia within the glomerulus and podocyte injury is central to pathogenesis [9].

We present three patients with MLCN. Two of them were associated with HIV infection while the third patient was treated with cisplatin-based chemotherapy.

## 2. Methods and Materials 

### 2.1. Case Number  1 

A 30-year-old African American male was admitted to the hospital who sustained a laceration a year ago for which he required stitches, and at that time he had some facial swelling. Physician suspected the fungus and antifungal was prescribed at that time. He was doing fine when he presented to our hospital with recurrent painful swelling and yellowish discharge. Comprehensive blood profile along with renal functions tests, CT orbits, and HIV testing were performed. He had an elevated BP and creatinine. HIV was positive and CT showed preseptal swelling. Skin biopsy came positive for coccidiosis. Nephrology consultation for suspected acute renal failure, with mild proteinuria (2.2 g/24 h), was done and a left renal needle biopsy was performed.

### 2.2. Case Number  2 

A 31-year-old African American male came to ER with complaints of progressive difficulty breathing for 4 months and unintentional weight loss of about 50 lbs. Chest X-ray was done which showed reticular nodular pattern. Infectious disease consultation was sought. Comprehensive blood profile along with renal functions tests and HIV testing were performed. He had an elevated BP and creatinine. HIV was positive. Nephrology consultation was done for an elevated creatinine (17 mg/dl) and proteinuria (3.7 g/24 h) with microscopic hematuria, and hemodialysis was initiated. CT scan showed bilateral ground-glass opacities. Bronchoalveolar lavage was done which confirmed* Pneumocystis carinii *pneumonia (PCP). A right renal needle biopsy was performed.

### 2.3. Case Number  3

A 65-year-old male with history of squamous cell carcinoma of tongue was being treated with cisplatin and radiation therapy. During the second round of chemotherapy he developed acute renal failure with oliguria and markedly elevated serum creatinine levels. The drug history was negative for nonsteroidal anti-inflammatory drugs (NSAIDs). Emergent renal biopsy was performed and appropriately triaged.

## 3. Results

HIV status for cases I and II was confirmed by ELISA and Western Blot tests.

Myeloma work-up included serum protein electrophoresis and immune-fixation confirmed absence of monoclonal gammopathy.

### 3.1. Renal Biopsy Interpretation

All two cases were diagnosed as myeloma-like cast nephropathy. In HIV positive patients biopsy finding confirmed HIV associated changes in addition to myeloma-like cast nephropathy. In all two cases immunofluorescence microscopy failed to demonstrate monoclonal restrictions.

### 3.2. Light Microscopy

**Figure 1:**
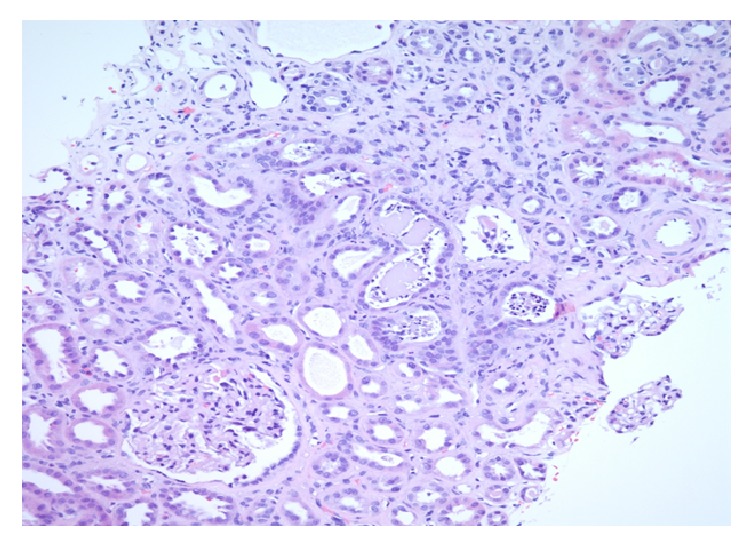
H&E section demonstrates fragments of fractured casts with neutrophilic reaction in a tubule. The glomeruli are unremarkable.

**Figure 2:**
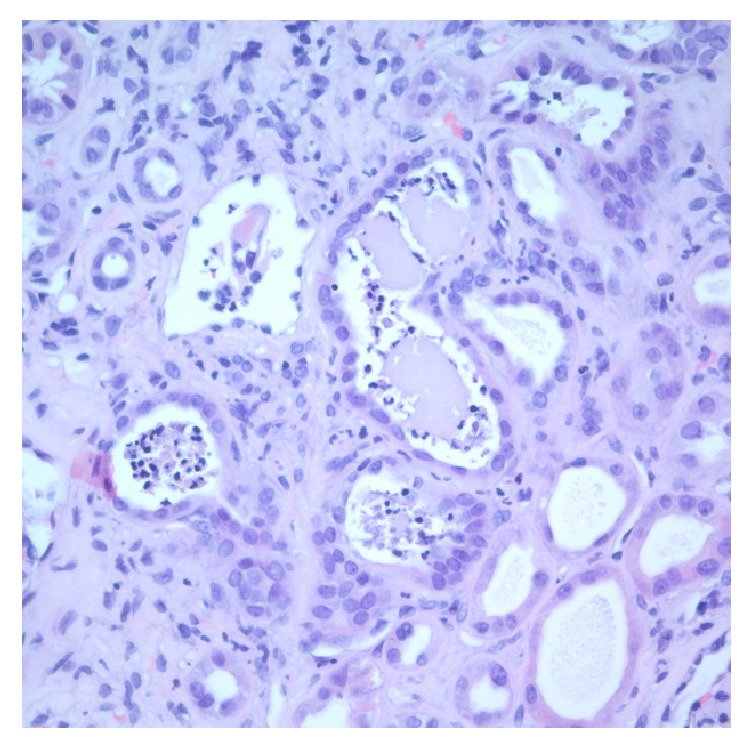
Many tubules are dilated with flattening of epithelium. In the adjacent tubules, multiple neutrophilic casts are seen.

Renal needle biopsy was done showing conventional HIV associated nephropathy with coexisting myeloma-like cast nephropathy. Light microscopy revealed foci of tubular cystic dilatation and diffuse tubular dilatation containing eosinophilic, Periodic Acid–Schiff (PAS) negative casts coupled with segmental collapse of capillary tufts and focal glomerulosclerosis. Multiple casts have intense neutrophilic reaction (Figures 1 and 2). First case has a diffuse focally dense interstitial inflammatory infiltrate including lymphocytes, plasma cells, neutrophils, and rare eosinophils, while second case has focal interstitial infiltrate including lymphocytes and rare neutrophils.

### 3.3. Electron Microscopy

**Figure 3:**
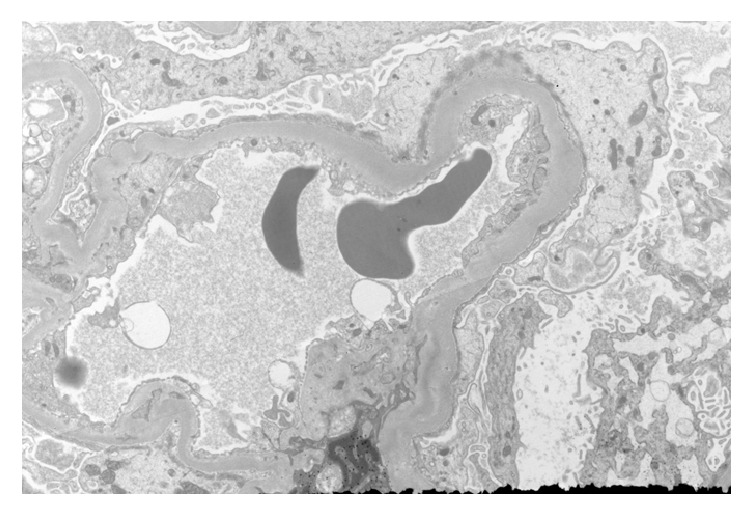
Electron micrograph of a glomerular capillary shows thickened glomerular basement membrane and diffuse foot process effacement.

**Figure 4:**
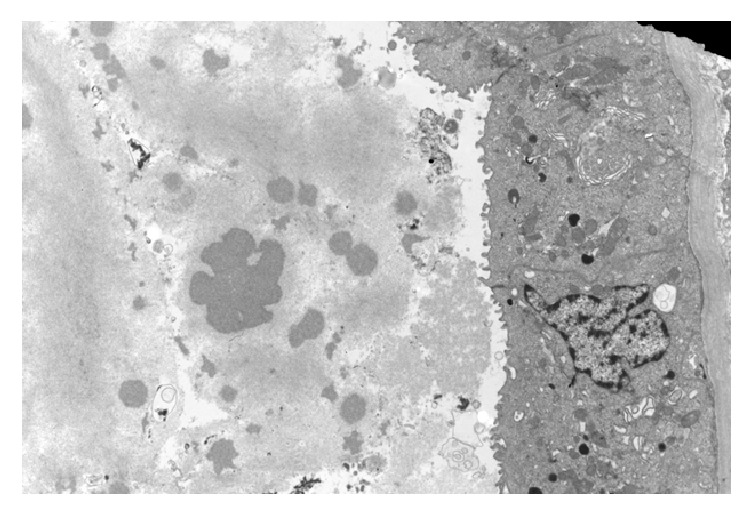
Electron micrograph of portion of cast shows light and dark areas with fine granular appearance. The tubular basement membrane seems laminated.

The ultrastructural findings include podocytes with extensive foot process effacement (Figure 3). The mesangial areas were slightly expended by increased matrix and mesangial cell processes. The glomerular basement membrane was slightly thickened. No well-defined immune complex type or organized protein deposits are identified. Endothelial cells were unremarkable and no tubule-reticular inclusions were identified in cytoplasm of glomerular endothelial cells. The tubular epithelial cells are low cuboidal with luminal cast of variable density. Tubular basement membrane exhibits vague lamination (Figure 4).

### 3.4. Immunofluorescence Microscopy

Direct immunofluorescence stains were performed using a panel of ten antisera. It failed to demonstrate monoclonal restriction. All glomeruli were negative for albumin, fibrinogen, C1q, C3, C4. IgA, IgM, kappa, and lambda light chain stains.

## 4. Discussion

Renal disease is one of the major causes of morbidity and mortality in HIV infected patients receiving effective antiretroviral treatment. HIVAN, the classic kidney disease of HIV infection and the most common cause of CKD in HIV infected individuals, is mostly seen in African American descent and is consistent with a strong genetic predisposition. HIV mediates dysregulation of glomerular podocytes, the epithelial cells that maintain the glomerular basement membrane, and apoptosis of renal tubular cells. The resulting lesion of HIVAN is a focal glomerulosclerosis (FGS) and microcytic dilation of the tubules filled with PAS positive casts [10, 11]. HIVAN usually presents as nephritic range proteinuria with a progressive loss of renal function and an interval of <10 months from the time of diagnosis to progression to ESRD [12–16]. Many reports suggest that black race [12–20], Haitian background [21], male gender, injection drug use [12–18, 21], and decreased CD4 cell count [21] are risk factors for the development of HIVAN. Interpretation of these reports is limited by the unknown effects of selection bias introduced through the methods by which patients were identified for inclusion. Several studies have included only those patients who were seen by nephrologists during admissions to the hospital for other diagnoses [17, 20, 22].

It appears that HIVAN occurs in the setting of specific host genes and it has been widely accepted that the gene encoding a non-myosin A heavy chain (MYH9) is associated with FGS, HIVAN, and end-stage kidney disease (ESKD) due to hypertension in individuals of African descent [23, 24]. The interaction of HIV with renal cells has not been clarified. Renal cells lack the classic receptors for HIV, CD4, and the chemokine receptors CCR5 and CXCR4. However, HIV-mRNA has been detected in renal epithelial cells, and HAART slows progression of HIVAN to renal failure [25]. Nonconventional receptors on renal cells such as C-type lectins including DC-Sign and lipid rafts have been suggested as portals of entry of HIV into renal cells [26]. Renal cell infection by HIV, however, is nonproductive [27].

The MLCN, as described earlier, has similar histomorphological features to MCN and both can lead to renal damage. CD4, a receptor for HIV-1 on lymphocyte and macrophages, is the main culprit involved in the pathogenesis of renal cast formation. The renal lesion, MLCN, caused by HIV-1 bears a similarity to the nephropathy seen in cases of myeloma kidney, and it is also quite similar to the lesions seen in experimental Bence Jones protein nephropathy [28]. The lesions are characterized intratubular protein cast formation that are weakly positive for PAS, associated with multinucleated giant cells and neutrophils mostly affecting the cortical collecting ducts, though the distal convoluted tubules are also seen to be affected. It has also been suggested in some experimental models that the targeted expression of viral genes in lymphocytes and lymphoid tissue recapitulates some of the pathologic findings that are observed in the Tg26 mouse model and in human HIVAN, suggesting that HIV-1 gene expression may play a role in the development of HIVAN in both renal and nonrenal tissues [29].

Cisplatin, a chemotherapeutic agent that has been well recognized as a nephrotoxic agent, primarily causes tubulointerstitial lesions and in spite of extreme efforts of finding less toxic alternatives, cisplatin yet is prescribed widely. The nephrotoxicity related to cisplatin involves multiple pathways including apoptotic cascades activation, endonucleases, and oxidant stress. Most of these pathways share the same mechanisms for the cytotoxic effects of cisplatin on neoplastic cells; hence the strategies to reduce the nephrotoxic effects of cisplatin may reduce the antineoplastic effects of cisplatin [30]. Despite all side effects and development of new drugs, its efficacy against neoplastic cells is vital and is still part of many chemotherapeutic regimens because its benefits outweigh the side effects.

Cisplatin is cleared by the kidney by both glomerular filtration and tubular secretion and its concentrations within the kidney exceed those in blood suggesting an active accumulation of drug by renal parenchymal cells. It damages the proximal tubules, specifically the S3 segment of the outer medullary stripe, while the mitochondrial swelling and nuclear pallor occur in the distal nephron. The glomerulus has no obvious morphologic changes. The site of injury involves both the distal tubule and collecting ducts or the proximal and distal tubules. The predominant lesion in patients with acute renal failure is acute necrosis and is located mostly in the proximal convoluted tubules. The severity of necrosis is dependent on dose, concentration, and duration. Patients with chronic nephrotoxicity have focal acute tubular necrosis characterized by cystic dilated tubules lined by a flattened epithelium showing atypical nuclei and atypical mitotic figures with hyaline casts. Long-term cisplatin treatment and injury may cause cyst formation and interstitial fibrosis. Two different membrane transporters capable of transporting cisplatin are Ctr1, a copper transporter, and OCT2, an organic cation transporter. Downregulation of Ctr1 expression in kidney decreases both cisplatin uptake and cytotoxicity [31], while loss of the OCT2 gene reduces urinary cisplatin excretion leading to nephrotoxicity [6].

## 5. Conclusion

The hallmark of MLCN histologically is the presence of tubular cast associated with intense inflammatory response. Although this is not a typical presentation of an acute renal failure caused by HIV infection, MLCN could be a form of renal injury seen on HIV patients. Based on the histologic evaluation of the two cases we presented, we noticed remarkable similarities in the histologic features of typical myeloma cast nephropathy and myeloma-like cast nephropathy, despite using the standard diagnostic tools to rule out the presence of monoclonal gammopathy or any other condition that could cause MLCN. The cause of the underlying pathology that is causing kidney damage could be obscured, and an incorrect diagnosis of MLCN can set the treating physician on the wrong path seeking diagnosis. Accordingly, having MLCN on the differential diagnosis list of typical myeloma cast nephropathy is advisable.
